# Supplementary material for: Combining agent-based, trait-based and demographic approaches to model coral-community dynamics
Source: eLife. 2020 Jul 23;9:e55993. doi: 10.7554/eLife.55993 (PMC7473774; doi:10.7554/eLife.55993)
Supplement: Transparent reporting form [file elife-55993-transrepform.pdf]

eLIFE
1st Floor
24 Hills Road
Cambridge CB2 1JP, UK
P 01223 855340
W elifesciences.org
T @elife

eLife's transparent reporting form

We encourage authors to provide detailed information *within their submission* to facilitate the interpretation and replication of experiments. Authors can upload supporting documentation to indicate the use of appropriate reporting guidelines for health-related research (see EQUATOR Network), life science research (see the BioSharing Information Resource), or the ARRIVE guidelines for reporting work involving animal research. Where applicable, authors should refer to any relevant reporting standards documents in this form.

If you have any questions, please consult our Journal Policies and/or contact us: editorial@elifesciences.org.

Sample-size estimation

- You should state whether an appropriate sample size was computed when the study was being designed
- You should state the statistical method of sample size computation and any required assumptions
- If no explicit power analysis was used, you should describe how you decided what sample (replicate) size (number) to use

Please outline where this information can be found within the submission (e.g., sections or figure legends), or explain why this information doesn't apply to your submission:

This information does not apply to our submission because our manuscript is a description of a model parameterization, calibration, validation and global sensitivity analysis. We did not conduct an experiment or sampled field data to answer a research question.

We replicated each simulation five times in the model calibration (Appendix S3) and the hierarchically structured validation (Appendix S5) to account for the stochasticity implemented in the model. We estimated that five was an appropriate number considering the computation time and variability of the results we observed from preliminary simulations.

In the global sensitivity analysis, we chose sample sizes and number of replicates (n = 1) according to the efficient protocol of Prowse et al., (2016); (Appendix S6: §2.5 and in: §1.5).

The statistical model predictions we did were constrained by data availability:
- imputation of missing trait data (Appendix S1: §2);
- initialization of colony size distributions (Appendix S2: §1);
- beta-regressions to predict species-specific index of bleaching susceptibility (Appendix S4: §1);
- generalized linear mixed effects model to predict bleaching probability as a function of degree-heating week (Appendix S4: §2).

Replicates

- You should report how often each experiment was performed
- You should include a definition of biological versus technical replication
- The data obtained should be provided and sufficient information should be provided to indicate the number of independent biological and/or technical replicates

- If you encountered any outliers, you should describe how these were handled
- Criteria for exclusion/inclusion of data should be clearly stated
- High-throughput sequence data should be uploaded before submission, with a private link for reviewers provided (these are available from both GEO and ArrayExpress)

Please outline where this information can be found within the submission (e.g., sections or figure legends), or explain why this information doesn't apply to your submission:

- We replicated each simulation five times in the model calibration (Appendix S3) and the hierarchically structured validation (Appendix S5);
- Definition of biological versus technical replication: not applicable
- Datasets and scripts are provided and are organized to facilitate reproducibility (http://dx.doi.org/10.17605/OSF.IO/CTQ43).
- Outliers: not applicable
- Criteria for exclusion/inclusion of datasets: not applicable
- High-throughput sequence data: not applicable

Statistical reporting

- Statistical analysis methods should be described and justified
- Raw data should be presented in figures whenever informative to do so (typically when N per group is less than 10)
- For each experiment, you should identify the statistical tests used, exact values of N, definitions of center, methods of multiple test correction, and dispersion and precision measures (e.g., mean, median, SD, SEM, confidence intervals; and, for the major substantive results, a measure of effect size (e.g., Pearson's r, Cohen's d)
- Report exact p-values wherever possible alongside the summary statistics and 95% confidence intervals. These should be reported for all key questions and not only when the p-value is less than 0.05.

Please outline where this information can be found within the submission (e.g., sections or figure legends), or explain why this information doesn't apply to your submission:

Thorough each document (manuscript and appendices), we attempted to place all the relevant and necessary information either on the figures or in their captions. We added tables following the figures when some of the information was too large to be placed in the caption (e.g., statistical model parameter estimates and their significance).

(For large datasets, or papers with a very large number of statistical tests, you may upload a single table file with tests, Ns, etc., with reference to sections in the manuscript.)

Group allocation

- Indicate how samples were allocated into experimental groups (in the case of clinical studies, please specify allocation to treatment method); if randomization was used, please also state if restricted randomization was applied
- Indicate if masking was used during group allocation, data collection and/or data analysis

Please outline where this information can be found within the submission (e.g., sections or figure legends), or explain why this information doesn't apply to your submission:

Not applicable; we did not conduct an experiment; we are presenting a model and its analysis.

Additional data files ("source data")

- We encourage you to upload relevant additional data files, such as numerical data that are represented as a graph in a figure, or as a summary table
- Where provided, these should be in the most useful format, and they can be uploaded as "Source data" files linked to a main figure or table
- Include model definition files including the full list of parameters used
- Include code used for data analysis (e.g., R, MatLab)
- Avoid stating that data files are "available upon request"

Please indicate the figures or tables for which source data files have been provided:

eLife Sciences Publications, Ltd is a limited liability non-profit non-stock corporation incorporated in the State of Delaware, USA, with company number 5030732, and is registered in the UK with company number FC030576 and branch number BR015634 at the address 1st Floor, 24 Hills Road, Cambridge CB2 1JP | August 2014

All our figures are reproduceable because we provide the entire datasets and scripts at http://dx.doi.org/10.17605/OSF.IO/CTQ43. Only Figure 1, Figure 2 and Appendix S5: Figure S3 are not reproduceable because they are illustrative and were not constructed from data.

We cannot provide the original datasets we used to calibrate the model because they are the property of the *Observatoire du Milieu Marin Martiniquais* (*OMMM*). These files can be requested via sharing agreement forms from Dr. Jean-Philippe Maréchal at marechal.jean@gmail.com. We provided a list of the original file names we used in the link mentioned above, in *Carturan_et_al_2020_project_files / Empirical_datasets_Martinique_calibration / Datasets_original / Readme.docx*.
